# Myositis as a rare complication after tocilizumab treatment

**DOI:** 10.1186/1546-0096-12-S1-P345

**Published:** 2014-09-17

**Authors:** Estefania Quesada-Masachs, Consuelo Modesto Caballero

**Affiliations:** 1 Pediatric Rheumatology, Hospital Universitari Vall d'Hebron, Barcelona, Spain

## Introduction

Tocilizumab (TCZ) is an anti-interleukin-6 receptor antibody. It has been accepted as a biological treatment in some subtypes of Juvenile Idiopathic Arthritis. Off-label it has been used (compassionate use) in some refractory pediatric autoimmune disorders. Despite being an effective treatment in most cases, this drug isn’t exempt of adverse events.

## Objectives

To describe a patient affected by an overlap autoimmune syndrome who presents an inflammatory myopathy as a rare complication during TCZ treatment.

## Methods

A 6-year-old boy affected by an overlap autoimmune syndrome characterized by facial skin lesions chilblain lupus like (histopathologically compatible with leukocytoclastic vasculitis), recurrent skin urticarial episodes and a severe lung disease (in the anatomopathological study is a linfoid intersticial neumonitis). He presents antinuclear antibodies positivity (maximum title reported 1/640), an undetermined antiDNA antibodies title (values around 31 UI/ml), no complement consumption of C3 neither C4, positives antiB2-glycoprotein and anticardiolipin IgG antibodies with persisting elevation of acute phase reactants highlighting an ESR permanently around 100 mm/h and thrombocytosis around 500x10^9 platelets. An extended auto-antibodies study was repeatedly performed without finding other auto-antibodies positivity. Several therapeutic strategies with different agents were previously tested (corticoids, azathioprine, hydroxychloroquine, anakinra, mycophenolate, gammaglobulin, tacrolimus). All of these therapies were discontinued because of inefficacy or lack of efficacy, and resolution of symptoms wasn’t achieved.

## Results

After a multidisciplinary evaluation TCZ treatment was initiated. After the first dose he presented improvement of general condition, of skin lesions and of the exertional dyspnea and a dramatical decrease in the acute phase reactants values. Two months later he referred subacute inflammatory pain in both lower limbs, predominantly proximal and symmetrical. The blood test revealed elevation of liver and muscular enzymes: alanine aminotransferase 91 UI/L, aspartate aminotransferase 59 UI/L, creatine kinase 1008 UI/L, aldolase 34.3 UI/L, lactate dehydrogenase 695 UI/L. A bilateral lower limb MRI was performed confirming enhanced STIR signal and edema affecting multiple muscles: right transverse abdominal muscle, bilateral gluteal muscles (maximus, medius and minimus), almost all muscle groups in both thighs respecting the intermedius and medialis vastus, the short and long adductors, and partially the magnus adductor. Inflammatory lesions of practically all muscle groups respecting the posterior tibial and soleus muscles in both legs were also observed. To sum up, a bilateral inflammatory myopathy was detected from abdomen to ankles. Intravenous gammaglobulin 1mg/kg/14 days treatment was introduced and TCZ discontinued achieving a fast improving in pain, function and normalizing blood parameters related with muscle inflammation. Two months later the myositis episode was on clinical and biological remission but the autoimmune disease continues.

## Conclusion

We report a case of inflammatory myopathy attributed as a rare complication after Tocilizumab treatment. To our knowledge, this adverse event of Tocilizumab treatment wasn’t previously described.

## Disclosure of interest

None declared

